# The Risk of Rectal Temperature Measurement in Neutropenia

**DOI:** 10.5041/RMMJ.10501

**Published:** 2023-07-31

**Authors:** Judith Olchowski, Noa Zimhony-Nissim, Lior Nesher, Leonid Barski, Elli Rosenberg, Iftach Sagy

**Affiliations:** 1 Infectious Diseases Institute, Rambam Health Care Campus, Haifa, Israel; 2 Clinical Research Center, Soroka University Medical Center, Beer-Sheva, Israel; 3 Faculty of Health Sciences, Ben-Gurion University of the Negev, Beer-Sheva, Israel; 4 Internal Medicine Division, Soroka University Medical Center, Beer-Sheva, Israel; 5 Infectious Disease Institute, Soroka University Medical Center, Beer-Sheva, Israel

**Keywords:** Bacteremia, neutropenic fever, rectal thermometry

## Abstract

**Background:**

Avoiding rectal thermometry is recommended in patients with neutropenic fever. Permeability of the anal mucosa may result in a higher risk of bacteremia in these patients. Still, this recommendation is based on only a few studies.

**Methods:**

This retrospective study included all individuals admitted to our emergency department during 2014–2017 with afebrile (body temperature <38.3°C) neutropenia (neutrophil count <500 cells/microL) who were over the age of 18. Patients were stratified by the presence or absence of a rectal temperature measurement. The primary outcome was bacteremia during the first five days of index hospitalization; the secondary outcome was in-hospital mortality.

**Results:**

The study included 40 patients with rectal temperature measurements and 407 patients whose temperatures were only measured orally. Among patients with oral temperature measurements, 10.6% had bacteremia, compared to 5.1% among patients who had rectal temperature measurements. Rectal temperature measurement was not associated with bacteremia, neither in non-matched (odds ratio [OR] 0.36, 95% confidence interval [CI] 0.07–1.77) nor in matched cohort analyses (OR 0.37, 95% CI 0.04–3.29). In-hospital mortality was also similar between the groups.

**Conclusions:**

Patients with neutropenia who had their temperature taken using a rectal thermometer did not experience a higher frequency of events of documented bacteremia or increased in-hospital mortality.

## INTRODUCTION

Neutrophils are crucial myeloid innate immune cells capable of mounting a rapid and powerful response to microbial infections. Increased production and mobilization of neutrophils are critical, especially in infections of bacterial nature. Any condition that leads to a dampened neutrophil response may predispose the host to severe bacterial infections, sepsis, and death.^1^ The most common insult impeding the neutrophil response is neutropenia; among the major causes of neutropenia are iatrogenic conditions (e.g. chemotherapy in oncological settings).^2^,^3^ Hence, cancer patients who are treated with chemotherapy are at increased risk of developing neutropenic fever. According to the Infectious Diseases Society of America, neutropenia is defined as an absolute neutrophil count less than 500 cells/microL.^4^ Profound neutropenia is defined as an absolute neutrophil count <100 cells/microL. Fever is defined as one measurement of body temperature ≥38.3°C or of ≥38.0°C sustained over a one-hour period.^4^,^5^

Although neutropenic fever is a common complication during chemotherapy, most patients do not have an identifiable site of infection or documentation of a positive culture.^4^,^6^,^7^ Indeed, only in 20%–30% of febrile events was the site of infection or etiology identified. Bacteremia is a common complication, especially in the setting of profound neutropenia or when the neutropenia lasts more than one week. For up to one-quarter of patients, a bloodstream infection is documented, yet some studies have reported even higher rates.^7^–^9^

The 2010 guidelines of the Infectious Diseases Society of America recommend early empirical antibiotic therapy with an anti-pseudomonal agent for every patient with fever and neutropenia. In addition, avoidance of rectal thermometry is advised. The guideline is supported by a number of studies.^4^,^10^–^13^ The recommendation against rectal thermometry is based on the notion that patients with neutropenia are more sensitive to local perianal mucosal breakdown and hence bacteremia. Notably, this advice is directed specifically towards hematopoietic stem cell transplant recipients, and not to all patients with neutropenia. However, studies that investigated the use of rectal thermometry among patients with neutropenia, or secondary to the use of chemotherapy, are scarce.^14^

This study was aimed at assessing the risk posed by rectal thermometry for the development of bacteremia among patients hospitalized with neutropenia.

## METHODS

### Study Population and Setting

This population-based retrospective cohort study was conducted at Soroka University Medical Center in Israel. The medical center is the only tertiary center in southern Israel and serves a population of more than 700,000 residents of southern Israel. This study was approved by the ethics committee of our center.

Patients were included in the study if they were older than 18 years, were admitted to the hospital’s emergency department (ED) during 2014–2017 with a neutrophil count <500 cells/microL during the first 48 hours of admission, and for whom two sets of blood cultures had been drawn (each set including one aerobic and one anaerobic bottle) in the ED and on admission to the internal medicine ward or intensive care unit (ICU). The highest oral or rectal temperature measured during the ED visit, prior to the decision to admit or discharge each patient, was used. If both oral and rectal measurements were taken, only the rectal measurement was considered. Patients with a fever >38.3°C, regardless of temperature modality, were excluded from the study.

### Data Extraction

The following baseline patient data were extracted from the hospital’s electronic database: demographic characteristics, medical history, type of malignancy (hematological versus solid), and the use of taxanes (a chemotherapy regimen known to pose a high risk for neutropenic fever) at any time during the 14 days prior to index hospitalization.^15^ In addition, the following data were retrieved regarding each patient’s hospitalization period: the admitting unit (ICU versus non-ICU), Charlson comorbidity index, low functional status (defined as being bedridden or a diagnosis of dementia), laboratory tests (including complete blood count, chemistry, and blood culture results), and the use of vasopressors. Data on whether or not the patients had perianal infection or mucositis were also noted.

A relevant positive blood culture was defined as the presence of bacterial growth during the first five days of index hospitalization. Bacterial growth data were extracted for each set of blood cultures and then by pathogens that were contaminants versus those that could cause bacteremia. Bacteremia was defined as at least two consecutive positive blood cultures with the same organism or one positive culture with a pathogen, for example *Staphylococcus aureus*, *Listeria monocytogenes*, *Pseudomonas aeruginosa*, *Escherichia coli*, and/or *Klebsiella pneumoniae* (see Supplementary Table 1 for the complete list of pathogens). Bacterial growth was defined as a contamination if the pathogen was included in the National Healthcare Safety Network common commensal list (defined by the Centers of Disease Control) and found growing in only one blood specimen.^16^

**Table 1:** Demographic and Clinical Characteristics of Neutropenic Patients. Parameter

Parameter	Temperature Measurement		*P* Value
	Oral *(n*=407)	Rectal (*n*=40)	
Males, *n* (%)	180 (44.2)	14 (35.0)	0.261
Age at admission (years), mean±SD	59.78±18.58	65.91±20.18	0.049
Smoker, *n* (%)*	123 (30.3)	10 (25.0)	0.485
Temperature (°C), mean±SD	37.20±0.51	37.53±0.63	<0.001
MASCC, median (IQR)	10 (9–11)	9 (8–11)	0.025
Charlson index, median (IQR)	5 (2–7)	5 (3–7)	0.861
Charlson index >4, *n* (%)	227 (55.8)	24 (60.0)	0.607
Ischemic heart disease, *n* (%)	8 (2.0)	1 (2.5)	0.818
Diabetes mellitus, *n* (%)	51 (12.5)	7 (17.5)	0.372
Cerebrovascular accident, *n* (%)	4 (1.0)	0 (0)	0.529
Chronic kidney disease, *n* (%)	44 (10.8)	7 (17.5)	0.204
Dementia, *n* (%)†	4 (1.0)	7 (17.9)	<0.001
Bed-ridden, *n* (%)†	13 (3.2)	10 (25.6)	<0.001
Hematological tumors, *n* (%)*,†	215 (53.2)	21 (53.8)	0.94
Solid tumors, *n* (%)*,†	124 (30.8)	8 (20.5)	0.184
Mucositis, *n* (%)*,†	35 (8.7)	3 (7.7)	0.836
Perianal infections, *n* (%)†	9 (2.2)	1 (2.6)	0.893
Undergoing chemotherapy treatment, *n* (%)*,†	220 (54.6)	14 (36.8)	0.027
Undergoing treatment including taxanes, *n* (%)†	24 (5.9)	2 (5.1)	0.837
Suspected infection as reason for admission, *n* (%)*,†	186 (45.8)	21 (53.8)	0.351
Neutrophils, mean±SD (10^3^/microL)	0.23±0.15	0.26±0.16	0.275
White blood cells, mean±SD (10^3^/microL)	4.33±6.25	2.72±3.57	0.109
Hemoglobin, mean±SD (g/dL)	10.15±2.1	9.8±2.68	0.327
Platelets, mean±SD (10^3^/microL)	140.60±114.79	121.85±99.85	0.319
ICU during index hospitalization, *n* (%)	6 (1.5)	1 (2.5)	0.618
Duration of hospitalization, median (IQR)	3 (1–5)	2 (0–6)	0.502
Release from ED, *n* (%)	106 (26.0)	16 (40.0)	0.059

* Number of patients with missing data in oral group: Smoker (1); Hematological tumors (3); Solid tumors (4), Mucositis (5); Undergoing chemotherapy treatment (4); Suspected infection as reason for admission (1).

† Number of patients with missing data in rectal group: Dementia (1); Bed-ridden (1); Hematological tumors (1); Solid tumors (1); Mucositis (1), Perianal infections (2); Undergoing chemotherapy treatment (2); Undergoing treatment including taxanes (1); Suspected infection as reason for admission (1).

ICU, intensive care unit; IQR, interquartile ratio; MASCC, Multinational Association for Supportive Care in Cancer; *n*, number; SD, standard deviation.

The primary outcome was the presence of bacteremia due to a relevant pathogen, as described above. The secondary outcome was in-hospital mortality.

### Statistical Analysis

Data were expressed as mean±standard deviation (SD), median and interquartile range, or number and percentage. *T*-test, chi-square, and non-parametric tests were used to compare the characteristics of patients with oral and rectal temperature measurements. A matched 1:3 ratio cohort was computed using caliper 0.001 and greedy matching, using the maximal possible number of applicants matched to their first choice. The cohort was adjusted for age, sex, and the Multinational Association for Supportive Care in Cancer (MASCC) score. Forward stepwise logistic regression of the dependent variable was performed to assess the association of temperature acquisition modality with bacteremia and in-hospital mortality. Data were presented as odds ratios (OR) and confidence intervals (CI). Each set of covariates (demographic, medical history, laboratory, etc.) was entered as a separate block to the model. The final model was selected based on model goodness of fit using the *C*-statistic and plausible clinical explanation. The final model of the primary analysis was adjusted for current chemotherapy use, the type of malignancy, and the baseline function level. To address the imbalance between hospitalized patients versus those discharged from the ED, a sensitivity analysis of the primary and secondary outcomes, including only those hospitalized, was conducted. Data analysis was performed using SPSS version 25.0. Results were considered statistically significant for *P*<0.05.

## RESULTS

In total, 447 patients were included in this study. Of these, 407 had only oral temperature measurements (oral group) and 40 had at least one rectal temperature measurement (rectal group). Table 1 presents the demographic and clinical characteristics of the patients stratified by temperature measurement group. The rectal group was older than the oral group (mean age 65.91±20.18 versus 59.78±18.58, *P*=0.049) and in poorer functional condition, as reflected by a higher rate of dementia (17.9% versus 1%, *P*<0.001) and a higher proportion being bedridden (25.6% versus 3.2%, *P*<0.001). Patients in the rectal versus oral groups had similar rates of mucositis (7.7% versus 8.7%, *P*=0.836) and perianal infections (2.6% versus 2.2%, *P*=0.893). The median MASCC score of the rectal group was lower (9 versus 10, *P*=0.025), and their admission temperature was higher (37.53°C±0.63 versus 37.20°C±0.51, *P*< 0.001). Table 2 presents the primary and secondary outcomes, bacteremia, and in-hospital mortality, stratified by temperature measurement group. The number of bacteremias was higher in the oral group compared to the rectal group (8.1% versus 5%, *P*=0.485). Although in-hospital mortality was numerically higher in the oral group, the difference was not significant (oral group 16.5% versus rectal group 5%, *P*=0.056).

**Table 2:** Clinical Outcomes of Neutropenic Patients. Outcome, *n* (%)

Outcome, *n* (%)	Temperature Measurement		*P* Value
	Oral *(n*=407)	Rectal (*n*=40)	
Positive blood cultures*	43 (10.6)	2 (5.1)	0.276

Contaminated	9 (20.9)	0 (0)	0.463
Pathological†	34 (8.1)	2 (5)	0.485
Gram-positive blood cultures	6 (14)	1 (50)	0.177

Gram-negative blood cultures	28 (65)	1 (50)	0.895

*Pseudomonas* blood cultures	7 (16.3)	0 (0)	0.424

Vasopressors during hospitalization‡	2 (0.5)	1/32 (3.1)	0.102

Hospitalization in oncology ward	161 (39.6)	4 (10)	<0.001

Hospitalization in internal medicine department	151 (37.1)	22 (55)	0.027

Mortality during hospitalization	67 (16.5)	2 (5)	0.056

* Culture results were missing in 2 patients (oral group, 1; rectal group, 1).

† Of 34 pathological cultures in the oral group, 1 culture result was missing, and 1 culture result was counted twice since the cultures grew Gram-positive (*n*=1) and Gram-negative (*n*=2) bacteria.

‡ Data were missing in 8 of 40 patients included in the rectal group.

Table 3 presents the non-matched and matched multivariate logistic regression analyses for the primary outcome, the development of bacteremia during an index hospitalization. For the matched analysis, 37 patients from the rectal group were matched to 111 patients in the oral group. The age, gender, clinical variables, and MASCC scores were similar between the matched groups (see Supplementary Table 2). After controlling for current chemotherapy treatment, tumor type, and functional status, the rectal group was not associated with bacteremia, neither in the non-matched nor in the matched analysis (OR 0.36, 95% CI 0.07–1.77; OR 0.37, 95% CI 0.04–3.29, respectively). The secondary outcome for in-hospital mortality is shown in Table 4. The rectal group was not associated with in-hospital mortality in the non-matched and matched analysis (OR 0.35 95% CI 0.08–1.53; OR 0.34 95% CI 0.07–1.64, respectively). The sensitivity analysis, which included only hospitalized patients, is presented in Supplementary Table 3. Both bacteremia and in-hospital mortality were similar in the sub-group of hospitalized patients, regardless of the temperature measurement modality.

**Table 3:** Logistic Regression for Bacteremia Among Neutropenic Patients. Covariate

Covariate	Non-Matched		Matched	
	OR (95% CI)	*P* Value	OR (95% CI)	*P* Value
Rectal temperature measurement	0.360 (0.073–1.776)	0.210	0.378 (0.043–3.297)	0.378
Undergoing chemotherapy	0.742 (0.324–1.703)	0.482	0.260 (0.036–1.856)	0.179
Solid tumor	1.745 (0.757–4.024)	0.191	1.590 (0.224–11.273)	0.642
Poor function (bedridden/dementia)	3.925 (1.214–12.683)	0.022	4.234 (0.432–41.515)	0.215

CI, confidence interval; OR, odds ratio.

**Table 4:** Logistic Regression for In-Hospital Mortality among Neutropenic Patients.

Covariate	Non-Matched		Matched	
	OR (95% CI)	*P* Value	OR (95% CI)	*P* Value
Rectal temperature measurement	0.355 (0.082–1.534)	0.165	0.347 (0.073–1.647)	0.183
Admission age >60	1.76 (1.339–2.314)	<0.001	2.089 (1.249–3.495)	0.005
Suspected infection	0.779 (0.453–1.338)	0.365	1.227 (0.441–3.416)	0.696
Hematologic disease	0.598 (0.348–1.027)	0.062	0.541 (0.196–1.493)	0.236

CI, confidence interval; OR, odds ratio.

## DISCUSSION

This study found that temperature measured rectally was not associated with a higher risk to develop subsequent bacteremia or in-hospital mortality. Our results remained consistent in a matched multivariate analysis.

Avoiding rectal temperature measurements is considered common practice among patients with neutropenia, especially those receiving chemotherapy. This is based on the assumption that these patients have a more permeable perianal mucosa due to mucosal damage, and that this subjects them to infection via gastrointestinal translocation.^7^,^14^,^17^,^18^ This mechanism was deduced from mice models by Kutty et al.^19^ The recommendation to avoid rectal temperature measurement is based on the Infectious Diseases Society of America guidelines from 2010 and the guidelines aimed at prevention of opportunistic infection among hematopoietic stem cell transplant recipients.^4^,^17^ These guidelines oppose the use of rectal thermometers, to avoid mucosal damage and breakdown. Yet, high-quality data to support this recommendation are scarce. Moreover, no published studies targeted patients who developed neutropenia secondary to chemotherapy. In our cohort, 52% of the patients were treated with chemotherapy during the 14 days prior to the index hospitalization. A few studies investigated invasive gastroenterology in patients with neutropenic fever. Abu-Sbeih et al. reported that patients with neutropenia and thrombocytopenia who underwent endoscopic procedures were not at an increased risk of post-procedure infections.^20^ They described post-procedure fever in 1% of the patients. Levy et al. reported the development of bacteremia in only 2% of patients who underwent endoscopic ultrasound fine-needle aspiration of rectal and perirectal lesions.^21^

The patients of our cohort whose temperature was measured rectally were older and with poorer performance status, as evidenced by higher proportions diagnosed with dementia and bedridden. Therefore, it is plausible that their temperature was taken rectally due to poorer functional status and not because they were sicker than the patients whose temperature was only taken orally. Although being bedridden or with dementia was associated with bacteremia in the non-matched analysis, these results did not remain statistically significant in a matched multivariate analysis. The association between bedridden state and bacteremia is described in the literature.^22^,^23^

To assess whether rectal temperature measurement was associated with bacteremia due to breakdown of skin, we also extracted data of mucositis and anal infection. Both presented similarly in the two study groups. Ten of our patients were documented as having perianal infections. Morcos et al. reviewed various types of perianal conditions, their frequencies, and their management in febrile neutropenic patients.^24^ The small number of patients with anal infection in our cohort does not enable us to determine whether this outcome is more prevalent among patients whose temperature is measured rectally. Further research is needed on a larger sample. Nonetheless, positive blood culture rates and, more specifically, the type of pathogens were similar between the study groups. Hence, our results suggest that skin breakthrough and bacteremia due to rectal temperature measurement are not likely to increase the risk to develop bacteremia after rectal temperature measurement.

This study has several limitations. First, due to the observational nature, causality between temperature measurement method and clinical outcomes cannot be inferred. Second, only a relatively small number of patients had rectal temperature measurements; this may have decreased the likelihood of attaining a statistically significant difference. Third, there was a relatively small number of positive blood cultures. The number of blood cultures confirmed as pathological was 33 (8.1%) in the oral and 2 (5%) in the rectal group (*P*=0.485). Although there was a relatively small number of positive blood cultures, these numbers represent the real-life experience of a large tertiary center during four years of follow-up. Fourth, some variables were entered by physicians to medical records (e.g. physical examination of mucositis and anal infections in physical examination), and this may have influenced reliability. Fifth, we acknowledge that some blood cultures may be taken after temperature examination, which may alter the interpretation of our results. Finally, selection bias should be considered, because patients who have their temperature taken rectally tend to be at lower functional status (bedridden, for example) or hemodynamically unstable. Yet, as mentioned above, the type of temperature measurement was not associated with increased risk of bacteremia and in-hospital mortality in either the matched or the non-matched analysis. Notwithstanding the above limitations, we have shown that rectal temperature measurement is not associated with an increased risk to develop bacteremia or in-hospital mortality.

Beyond the contribution of our study to challenging axioms and supporting the practice of evidence-based medicine, we believe that, by not avoiding rectal temperature measurements in neutropenic patients, early detection and diagnosis of fever and hence infections can be achieved. Early recognition and diagnosis of sepsis, especially in neutropenic patients, will lead to early directed therapy. It has been established^25^ that early administration of antibiotics is crucial for the treatment of sepsis and has an impact on mortality rates. Hopefully, early diagnosis will lead to early treatment, shorter hospitalizations, and lower mortality rates. Future studies should also attend to this issue, to improve the quality of care of patients with neutropenic fever.

## Supplementary Information



## Abbreviations

CI: confidence interval
ED: emergency department
ICU: intensive care unit
MASCC: Multinational Association for Supportive Care in Cancer
OR: odds ratio
