# Supplementary material for: The Risk of Rectal Temperature Measurement in Neutropenia
Source: Rambam Maimonides Med J. 2023 Jul 31;14(3):e0014. doi: 10.5041/RMMJ.10501 (PMC10393468; doi:10.5041/RMMJ.10501)
Supplement: Supplementary file 1 [file rmmj-14-3-e0014AM.docx]

This appendix has been provided by the authors for the benefit of readers

Supplement to The Risk of Rectal Temperature Measurement in Neutropenia

Olchowski J, Zimhony-Nissim N, Nesher L, Barski L, Rosenberg E, Sagy I. The Risk of Rectal Temperature Measurement in Neutropenia. Rambam Maimonides Med J 2023;11(3):e0014. doi:10.5041/RMMJ.10501

Supplementary tables to the above cited manuscript are presented below and on the following pages.

Supplementary Table 1. Bloodstream Infection Pathogens Detected and the Probable Contaminants.

| **Confirmed Bloodstream Infection - Pathogen** | **Probable Contaminant** |
| --- | --- |
| *Enterococcus faecium* (1) | *Staphylococcus*, coagulase-negative (6) |
| *Escherichia coli* (10) | *Corynebacterium* (1) |
| *Staphylococcus*, coagulase-negative (1 in more than 2 consecutive cultures) | *Micrococcus* (1) |
| *Pseudomonas aeruginosa* (7) | *Streptococcus gallolyticus* group (1) |
| *Staphylococcus aureus* (4 in total, 1 of them MRSA) |  |
| *Klebsiella* *pneumoniae* (10) |  |
| *Moraxella osloensis* (1) |  |
| *Streptococcus* *pneumoniae* (1) |  |
| *Streptococcus pyogenes* (1) |  |
| *Citrobacter koseri* (1) |  |
| *Providencia stuartii* (1) |  |
| *Clostridium difficile* (1) |  |
| *Campylobacter* (1) |  |

Supplementary Table 2. Demographic and Clinical Characteristics of the Matched Cohort.

| **Parameter** | **Oral Measurement (***n***=111)** | **Rectal Measurement (***n***=37)** | *P* **Value** |
| --- | --- | --- | --- |
| Males, *n* (%) | 51 (45.9) | 14 (37.8) | 0.389 |
| Age at admission (years), mean±SD | 61.04±19.09 | 64.12±19.85 | 0.402 |
| Smoker, *n* (%) | 30 (27.0) | 10 (27.0) | 0.977 |
| Temperature (°C), mean±SD | 37.13±0.48 | 37.49±0.64 | 0.003 |
| MASCC, median (IQR) | 10 (9-11) | 9 (8-11) | 0.142 |
| CCI, median (IQR) | 5 (2-7) | 4 (2-6) | 0.302 |
| Charlson index >4, *n* (%) | 65 (58.6) | 21 (56.8) | 0.847 |
| Ischemic heart disease, *n* (%) | 1 (0.9) | 1 (2.7) | 0.411 |
| Diabetes mellitus, *n* (%) | 16 (14.4) | 6 (16.2) | 0.793 |
| Cerebrovascular accident, *n* (%) | 0 (0) | 0 (0) | 1.000 |
| Chronic kidney disease, *n* (%) | 15 (13.5) | 5 (13.5) | 1.000 |
| Dementia, *n* (%) | 1 (0.9) | 6 (16.2) | <0.001 |
| Bedridden, *n* (%)^†^ | 1 (0.9) | 10 (27.8) | <0.001 |
| Hematological tumors, *n* (%)^†^ | 59 (53.2) | 21 (58.3) | 0.623 |
| Solid tumors, *n* (%)*^,†^ | 35 (32.1) | 8 (22.2) | 0.273 |
| Mucositis, *n* (%) | 9 (8.1) | 3 (8.1) | 0.977 |
| Perianal infections, *n* (%) | 3 (2.7) | 1 (2.7) | 0.987 |
| Current chemotherapy treatment, *n* (%)*^,†^ | 57 (51.8) | 14 (38.9) | 0.178 |
| Currently treated, including taxanes, *n* (%)^†^ | 7 (6.3) | 2 (5.6) | 0.861 |
| Suspected infection as a reason for admission, *n* (%)*^,†^ | 47 (43.1) | 19 (52.8) | 0.293 |
| NEU, mean±SD (10^3^/microL) | 0.23±0.15 | 0.26±0.15 | 0.335 |
| WBC, mean±SD (10^3^/microL) | 4.5±6.41 | 2.6±3.57 | 0.025 |
| HGB, mean±SD (g/dL) | 10.19±2.01 | 9.66±2.61 | 0.209 |
| PLT, mean±SD (10^3^/microL) | 125.48±95.25 | 116.5±95.25 | 0.621 |
| Composite outcome, *n* (%) | 29 (26.1) | 9 (24.3) | 0.828 |
| ICU during index hospitalization, *n* (%) | 1 (0.9) | 1 (2.7) | 0.411 |
| Duration of hospitalization, median (IQR) | 3 (1-4) | 2 (0-5) | 0.231 |
| Hospitalization longer than one week, *n* (%) | 11 (9.9) | 6 (16.2) | 0.297 |
| Release from ED, *n* (%) | 26 (23.4) | 16 (43.2) | 0.021 |
| Positive blood cultures, *n* (%) | 6 (5.4) | 2 (5.4) | 1.000 |
| Blood cultures confirmed as pathological, *n* (% out of positive cultures) | 4 (66.7) | 2 (100) | 0.396 |
| Contaminated blood cultures, *n* (% out of positive cultures) | 2 (33.3) | 0 (0) | 0.346 |
| Gram positive blood cultures, *n* (% out of pathological cultures) | 1 (25) | 1 (50) | 0.346 |
| Gram-negative blood cultures, *n* (% out of pathological cultures) | 3 (75) | 1 (50) | 1.000 |
| *Pseudomonas* blood cultures, *n* (% out of Gram-negative cultures) | 1 (33.3) | 0 (0) | 0.537 |
| Vasopressors during hospitalization, *n* (%) | 0 (0) | 0 (0) | 1.000 |
| Hospitalization in oncology ward, *n* (%) | 47 (42.3) | 4 (10.8) | <0.001 |
| Hospitalization in internal medicine department, *n* (%) | 38 (34.2) | 19 (51.4) | 0.064 |
| Mortality during hospitalization, *n* (%) | 18 (16.2) | 2 (5.4) | 0.096 |
| 30-Day mortality, *n* (%) | 2 (1.8) | 2 (5.4) | 0.242 |

* Number of patients with missing data in oral group: Solid tumors (2); Current chemotherapy treatment (1); Suspected infection as a reason for admission (2).

^†^ Number of patients with missing data in rectal group: Bedridden (1); Hematological tumors (1); Solid tumors (1); Current chemotherapy treatment (1); Currently treated, including taxanes (1); Suspected infection as a reason for admission (1).

CCI, Charlson comorbidity index; Composite outcome: ICU admission, in-hospital mortality, administration of vasopressors, hospitalization >7 days; ED, emergency department; HGB, hemoglobin; ICU, intensive care unit; IQR, interquartile range; SD, standard deviation; MASCC, Multinational Association for Supportive Care in Cancer; NEU, neutrophils; PLT, platelets; WBC, white blood cells.

Supplementary Table 3. Multivariate Sensitivity Analysis Including Only Hospitalized Patients, by Bacteremia and In-hospital Mortality.

| Model | Covariate | OR (95% CI) | *P* Value |
| --- | --- | --- | --- |
| Bacteremia | Rectal vs. oral temperature measurement | 0.608 (0.108-3.433) | 0.573 |
|  | Current chemotherapy | 0.727 (0.268-1.970) | 0.531 |
|  | Solid tumor | 2.488 (0.944-6.556) | 0.065 |
|  | Poor function (bedridden/dementia) | 3.392 (0.861-13.364) | 0.081 |
| In-hospital mortality | Rectal vs. oral temperature measurement | 0.411 (0.051-3.290) | 0.402 |
|  | Admission age >60 years | 1.795 (1.280-2.516) | 0.001 |
|  | Suspected infection | 0.644 (0.325-1.277) | 0.208 |
|  | Hematologic disease | 0.342 (0.172-0.681) | 0.002 |
